# Retinal age gap as a predictive biomarker of stroke risk

**DOI:** 10.1186/s12916-022-02620-w

**Published:** 2022-11-30

**Authors:** Zhuoting Zhu, Wenyi Hu, Ruiye Chen, Ruilin Xiong, Wei Wang, Xianwen Shang, Yifan Chen, Katerina Kiburg, Danli Shi, Shuang He, Yu Huang, Xueli Zhang, Shulin Tang, Jieshan Zeng, Honghua Yu, Xiaohong Yang, Mingguang He

**Affiliations:** 1 Department of Ophthalmology, Guangdong Academy of Medical Sciences, Guangdong Provincial People’s Hospital, Guangzhou, China; 2 Centre for Eye Research Australia, Ophthalmology, University of Melbourne, Melbourne, Australia; 3 Ophthalmology, Department of Surgery, University of Melbourne, Melbourne, Australia; 4 State Key Laboratory of Ophthalmology, Zhongshan Ophthalmic Center, Sun Yat-sen University, Guangzhou, China; 5 John Radcliffe Hospital, Oxford University Hospitals NHS Foundation Trust, Oxford, UK

**Keywords:** Retinal age, Stroke, Prediction, Biomarker

## Abstract

**Background:**

The aim of this study is to investigate the association of retinal age gap with the risk of incident stroke and its predictive value for incident stroke.

**Methods:**

A total of 80,169 fundus images from 46,969 participants in the UK Biobank cohort met the image quality standard. A deep learning model was constructed based on 19,200 fundus images of 11,052 disease-free participants at baseline for age prediction. Retinal age gap (retinal age predicted based on the fundus image minus chronological age) was generated for the remaining 35,917 participants. Stroke events were determined by data linkage to hospital records on admissions and diagnoses, and national death registers, whichever occurred earliest. Cox proportional hazards regression models were used to estimate the effect of retinal age gap on risk of stroke. Logistic regression models were used to estimate the predictive value of retinal age and well-established risk factors in 10-year stroke risk.

**Results:**

A total of 35,304 participants without history of stroke at baseline were included. During a median follow-up of 5.83 years, 282 (0.80%) participants had stroke events. In the fully adjusted model, each one-year increase in the retinal age gap was associated with a 4% increase in the risk of stroke (hazard ratio [HR] = 1.04, 95% confidence interval [CI]: 1.00–1.08, *P* = 0.029). Compared to participants with retinal age gap in the first quintile, participants with retinal age gap in the fifth quintile had significantly higher risks of stroke events (HR = 2.37, 95% CI: 1.37–4.10, *P* = 0.002). The predictive capability of retinal age alone was comparable to the well-established risk factor-based model (AUC=0.676 vs AUC=0.661, *p*=0.511).

**Conclusions:**

We found that retinal age gap was significantly associated with incident stroke, implying the potential of retinal age gap as a predictive biomarker of stroke risk.

**Supplementary Information:**

The online version contains supplementary material available at 10.1186/s12916-022-02620-w.

## Background

Stroke is the second leading cause of death and a leading cause of disability worldwide [1, 2]. An accurate prediction of stroke risk is of great importance in identifying individuals at high risks at an early stage to implement personalized preventative and therapeutic interventions [3]. Chronological age is one of the most important risk factors for stroke [4]. Of note, the trajectories of ageing vary significantly among individuals with the same chronological age [5]. Growing evidence has shown that biological age, an integrated measurement reflecting the combined effects of environmental, lifestyle and genetic factors, could have more value than chronological age in the prediction of age-related diseases and mortality [6, 7].

This highlights the need for an accurate biomarker of biological ageing. A series of effective measurements have been developed by previous studies [8–11]. Notably, increased biological age measured by leukocyte telomere length, epigenetic clock or brain age has been associated with a higher risk of stroke [12–15]. However, these measurements are invasive, expensive and time-consuming, and therefore, not suitable for widespread use.

The retina is considered as an extension of the central nervous system (CNS), offering a unique “window” that can non-invasively reflect the changes of brain neural tissues and vasculature in vivo [16]. Mounting evidence has reported that various retinal parameters (such as tortuosity, fractal dimension) [17], retinal pathologies (such as arteriovenous nicking and microaneurysms) [18] or retinal diseases (such as diabetic retinopathy, retinal artery or vein occlusion) [19–21] were associated with the risk of cerebrovascular diseases such as stroke. This might be explained by the similarities between the retina and the brain in terms of embryological origins, anatomy and physiology [22]. Therefore, the retina could potentially be used to measure systemic senescence. With the advances of deep learning (DL) technology in analyzing medical images [23], we have developed an algorithm that uses a retinal image as the input to predict the retinal age of an individual accurately. We also validated the retinal age gap, determined as retinal age derived from fundus images minus chronological age, was a robust biomarker associated with the risk of mortality. However, whether the retinal age gap can be used as a biomarker and/or a predictor of incident stroke remains unknown. Therefore, we aimed to investigate the association between the retinal age gap and the risk of incident stroke, and its predictive capability of stroke based on data from the UK Biobank study.

## Methods

### Study population

The UK biobank is a large-scale population-based prospective cohort study of 502, 656 UK residents aged 40 to 69 years who were registered with the National Health Service (NHS). The overall study protocols and data are available elsewhere [24]. Briefly, baseline assessments were performed between 2006 and 2010 in 22 assessment centres across the UK. Participants completed electronic questionnaires to provide information on socio-demographics, lifestyle, environmental exposures, medical history and cognitive functions. Physical examinations including blood pressure, heart rate, grip strength, anthropometrics and spirometry were done for all participants. Biological samples including stored blood, urine and saliva samples were collected. Follow-up of medical conditions was performed mainly through data linkages to hospital records and mortality registries.

This study was reviewed and approved by the National Information Governance Board for Health and Social Care and the NHS North West Multicenter Research Ethics Committee (11/NW/0382) and the Biobank consortium (application no. 62489). Since we used de-identified data in a public dataset, the Medical Research Ethics Committee of Guangdong Provincial People’s Hospital waived the requirements to obtain the ethical approval. The study was performed in accordance with the Declaration of Helsinki. All participants provided informed consent.

### Fundus photography

Between 2009 and 2010, ophthalmic examinations were introduced at six assessment centres across the UK [25]. The 45° non-mydriatic retinal fundus and optical coherence tomography (OCT) imaging of the optic disc and macular were captured using a spectral domain OCT for each eye (Topcon 3D OCT 1000 Mk2, Topcon Corp, Tokyo, Japan). At baseline, ophthalmic examinations were completed in 66,500 participants, resulting in a total of 131,238 fundus images.

### Deep learning model for age prediction

A total of 80,169 images from 46,969 participants passed the image quality check and were included in the analysis. The characteristics of the participants stratified by the number of images passing the quality check were described in detail in Additional file 1: Table S1. Among 46,969 participants, 11,052 participants did not report any previous disease at baseline. The DL model for age prediction was constructed based on fundus images of disease-free participants. To maximize the data available, binocular images, if available, were used for training and validation. The association between the retinal age gap and stroke was investigated using images from the remaining 35,304 participants who had no history of stroke at baseline. Images from the right eye were included in the test set to predict retinal age and images from the left eye were used only if images from the right eye were not available.

The methods of retinal age prediction using DL models were described in detail in a previous study [26]. Our previous study has assessed the performance of the DL model for age prediction. The DL model accurately predicted retinal age, as reflected by a strong correlation of 0.80 (*P*<0.001) between predicted retinal age and chronological age, as well as an overall mean absolute error (MAE) of 3.55 years. Attention maps retrieved from the DL model for age prediction mainly highlighted areas around the retinal vessels in the fundus images.

### Retinal age gap definition

The retinal age gap was defined as the difference between the retinal age predicted by the DL model based on fundus images and the chronological age. A positive retinal age gap indicated an ‘older’-appearing retina, while a negative one indicated a ‘younger’-appearing retina.

### Stroke ascertainment

Stroke was ascertained by the UK Biobank Outcome Adjudication Group, and was defined by codes 430.X, 431.X, 433.X1, 434.X1, 436.X in the 9th edition of the International Classification of Diseases (ICD-9) and ICD-10 codes I60, I61, I63, and I64. Stroke events were derived from linked electronic health records, including hospital records on admissions and diagnoses from hospitals in England, Scotland and Wales, as well as cause of death obtained from national death registers. The date of the first known stroke after baseline assessment was recorded. The follow-up period for each participant was defined from the recruitment date of the UK Biobank study to 28th February 2018 (the last follow-up date), or to the date of the first known stroke, whichever came first.

### Covariates

Covariates in the present analyses included socio-demographic factors (baseline age, sex, ethnicity, Townsend deprivation indices [TDI], education attainment), lifestyle factors (smoking status, drinking status, physical activity level), and general health status. Baseline age and sex were obtained from central registry or self-reported questionnaires. Self-reported ethnicity was divided into white or non-white. TDI was a proxy measure of socioeconomic status based on the postcode. Education attainment was classified into college/university degree or above, or others. Smoking and drinking status were categorized as current/previous users, or never. Physical activity level was categorized into reaching moderate/vigorous/walking recommendation or not. General health status was classified as excellent/good or fair/poor. Body mass index (BMI) was calculated as the weight of an individual in kilograms divided by their height in meters squared. Obesity was defined as a BMI of 30 kg/m^2^ or above. Diabetes mellitus was defined as any record of self-reported or doctor-diagnosed diabetes mellitus, or the use of anti-hyperglycaemic medications or insulin. Hypertension was defined as self-reported, or doctor-diagnosed hypertension, or the use of antihypertensive drugs, or an average systolic blood pressure ≥ 130 mmHg or an average diastolic blood pressure ≥ 80 mmHg.

### Statistical analyses

Continuous variables were reported as means and standard deviations (SDs), while categorical variable was reported as numbers and percentages. Unpaired t-tests and Chi-square tests were performed to examine the differences of the continuous and categorical variables, respectively. The log-rank test was used for comparing the survival distributions among different retinal age gap groups. Cox proportional hazards regression models were used to estimate the effect of retinal age gap on the risk of stroke. Each variable was assessed for the proportional hazards assumption and all of them met the assumption. Retinal age gap was introduced into the models as a continuous variable (per one-year increase) and a categorical variable (quintiles), respectively. Model I adjusted for baseline age, sex, and ethnicity. Model II adjusted for all variables in model I, and also TDI, educational level, smoking status, drinking status, physical activity level, diabetes mellitus, hypertension, obesity and general health status. Logistic regression models were used to estimate the predictive value of the well-established conventional risk factor-based model (including age, gender, smoking status, history of diabetes, systolic blood pressure, and total cholesterol to HDL-cholesterol ratio) [27] and the retinal age-based model in 10-year stroke risk. Area under the receiver-operating-characteristic curve (AUC) was used to describe the discrimination of the models in predicting 10-year stroke risk.

Sensitivity analysis was performed to adjust for the age-squared term in the final models in addition to age. We also investigated whether retinal age acceleration residual (defined as the residuals from regressing predicted retinal age against chronological age) was a biomarker of stroke in the second sensitivity analysis.

A two-sided *p* value of < 0·05 indicated statistical significance. All analyses were performed using R (version 3.3.0, R Foundation for Statistical Computing, www.R-project.org, Vienna, Austria) and Stata (version 13, StataCorp, TX, USA).

## Results

### Study sample

A total of 35,304 participants without any stroke history at baseline were included in the analyses (mean age 56.7 ± 8.04 years, 55.9% females and 93.2% white ethnicity). Table 1 depicts the baseline characteristics of the participants overall and stratified by retinal age gap quintiles. All features were significantly different across quintiles of retinal age gap, except for a history of diabetes mellitus.

**Table 1:** Baseline characteristics of the study participants and stratified by quintiles of retinal age gap

Baseline characteristics	Total	Retinal age gap					***P*** value
		Q1	Q2	Q3	Q4	Q5	
*N*	35,304	7061	7061	7061	7061	7060	-
Age, mean (SD), years	56.7 (8.04)	63.4 (4.59)	60.5 (6.02)	57.1 (6.84)	53.0 (7.39)	49.4 (6.18)	**<0.001**
Gender, No. (%)							
Female	19,730 (55.9)	3557 (50.4)	3929 (55.6)	3963 (56.1)	4135 (58.6)	4146 (58.7)	**<0.001**
Male	15,574 (44.1)	3504 (49.6)	3132 (44.4)	3098 (43.9)	2926 (41.4)	2914 (41.3)	
Ethnicity, No. (%)							
White	32,908 (93.2)	6652 (94.2)	6661 (94.3)	6612 (93.6)	6476 (91.7)	6507 (92.2)	**<0.001**
Others	2396 (6.79)	409 (5.79)	400 (5.66)	449 (6.36)	585 (8.28)	553 (7.83)	
Deprivation index, mean (SD)	−1.10 (2.95)	−1.46 (2.79)	−1.33 (2.81)	−1.10 (2.96)	−0.93 (3.02)	−0.68 (3.08)	**<0.001**
Education level, No. (%)							
College/university	12,306 (34.9)	2148 (30.4)	2291 (32.5)	2416 (34.2)	2629 (37.2)	2822 (40.0)	**<0.001**
Others	22,998 (65.1)	4913 (69.6)	4770 (67.5)	4645 (65.8)	4432 (62.8)	4238 (60.0)	
Smoking status, No. (%)							
Never	19,517 (55.5)	3826 (54.5)	3856 (54.8)	3817 (54.3)	3957 (56.3)	4061 (57.8)	**<0.001**
Former/current	15,614 (44.5)	3191 (45.5)	3176 (45.2)	3212 (45.7)	3069 (43.7)	2966 (42.2)	
Drinking status, No. (%)							
Never	1547 (4.40)	358 (5.08)	282 (4.00)	299 (4.24)	305 (4.34)	303 (4.31)	**0.025**
Former/current	33,652 (95.6)	6684 (94.9)	6768 (96.0)	6747 (95.8)	6726 (95.7)	6727 (95.7)	
Obesity, No. (%)							
No	26,203 (74.6)	5320 (75.8)	5291 (75.3)	5282 (75.2)	5195 (74.0)	5115 (72.8)	**<0.001**
Yes	8922 (25.4)	1699 (24.2)	1739 (24.7)	1747 (24.8)	1829 (26.0)	1908 (27.2)	
Meeting PA recommendation, No. (%)							
No	5212 (18.0)	886 (15.6)	967 (16.9)	1043 (18.1)	1100 (18.9)	1216 (20.5)	**<0.001**
Yes	23,706 (82.0)	4803 (84.4)	4748 (83.1)	4728 (81.9)	4713 (81.1)	4714 (79.5)	
History of diabetes, No. (%)							
No	33,026 (93.6)	6600 (93.5)	6605 (93.5)	6597 (93.4)	6644 (94.1)	6580 (93.2)	0.274
Yes	2278 (6.45)	461 (6.53)	456 (6.46)	464 (6.57)	417 (5.91)	480 (6.80)	
History of hypertension, No. (%)							
No	8624 (24.4)	1186 (16.8)	1403 (19.9)	1723 (24.4)	1984 (28.1)	2328 (33.0)	**<0.001**
Yes	26,680 (75.6)	5875 (83.2)	5658 (80.1)	5338 (75.6)	5077 (71.9)	4732 (67.0)	
General health status, No. (%)							
Excellent/good	24,562 (70.0)	5118 (72.8)	5051 (71.8)	4950 (70.5)	4783 (68.2)	4660 (66.5)	**<0.001**
Fair/poor	10,549 (30.0)	1916 (27.2)	1985 (28.2)	2074 (29.5)	2230 (31.8)	2344 (33.5)	

*SD* standard deviation, *PA* physical activity, *Q* quintile

### Incident stroke

During a median follow-up of 5.83 years (interquartile range [IQR]: 5.74–5.97), a total of 282 (0.80%) participants had stroke events. Table 2 shows the baseline characteristics of participants with and without incident stroke events. Participants who experienced a stroke were more likely to be older, of male gender, less educated, less physically active, obese, and with a history of diabetes mellitus and hypertension, and with a poorer general health status.

**Table 2:** Baseline characteristics stratified by incident stroke

Baseline characteristics	Non-stroke group	Stroke group	***P*** value
*N*	35,022	282	-
Age, mean (SD), years	56.7 (8.04)	62.0 (6.42)	**<0.001**
Gender, No. (%)			
Female	19,603 (56.0)	127 (45.0)	**<0.001**
Male	15,419 (44.0)	155 (55.0)	
Ethnicity, No. (%)			
White	32,641 (93.2)	267 (94.7)	0.325
Others	2381 (6.80)	15 (5.32)	
Deprivation index, mean (SD)	−1.10 (2.95)	−0.98 (3.09)	0.507
Education level, No. (%)			
College/university	12,226 (34.9)	80 (28.4)	**0.022**
Others	22,796 (65.1)	202 (71.6)	
Smoking status, No. (%)			
Never	19,375 (55.6)	142 (51.1)	0.132
Former/current	15,478 (44.4)	136 (48.9)	
Drinking status, No. (%)			
Never	1529 (4.38)	18 (6.38)	0.102
Former/current	33,388 (95.6)	264 (93.6)	
Obesity, No. (%)			
No	26,019 (74.7)	184 (66.0)	**0.001**
Yes	8827 (25.3)	95 (34.0)	
Meeting PA recommendation, No. (%)			
No	5159 (18.0)	53 (24.1)	**0.019**
Yes	23,539 (82.0)	167 (75.9)	
History of diabetes, No. (%)			
No	32,788 (93.6)	238 (84.4)	**<0.001**
Yes	2234 (6.38)	44 (15.6)	
History of hypertension, No. (%)			
No	8590 (24.5)	34 (12.1)	**<0.001**
Yes	26,432 (75.5)	248 (87.9)	
General health status, No. (%)			
Excellent/good	24,408 (70.1)	154 (55.4)	**<0.001**
Fair/poor	10,425 (29.9)	124 (44.6)	

*SD* standard deviation, *PA* physical activity

### Retinal age gap and stroke

As shown in Table 3, after adjusting for age, gender and ethnicity, each one-year increase in the retinal age gap was independently associated with a 5% increase in the risk of stroke (Hazard Ratio [HR] = 1.05, 95% confidence interval [CI]: 1.01–1.08, *P* = 0.006). This finding remained significant after further adjustments for other confounding factors (HR=1.04, 95% CI: 1.00–1.08, *P* = 0.029).

**Table 3:** Association between retinal age gap with incident of stroke

Retinal age gap	Model I		Model II	
	HR (95% CI)	***P*** value	HR (95% CI)	***P*** value
Retinal age gap, per one age (years)	1.05 (1.01–1.08)	**0.006**	1.04 (1.00–1.08)	**0.029**
Retinal age gap				
Q1	1 [Reference]	-	1 [Reference]	-
Q2	1.24 (0.90–1.70)	0.188	1.16 (0.80–1.69)	0.433
Q3	1.07 (0.74–1.57)	0.708	1.10 (0.72–1.70)	0.660
Q4	1.45 (0.95–2.22)	0.086	1.29 (0.78–2.14)	0.323
Q5	2.06 (1.26–3.36)	**0.004**	2.37 (1.37–4.10)	**0.002**

Model I adjusted for age, gender, and ethnicity

Model II adjusted for covariates in Model I+deprivation, education level, smoking status, drinking status, obesity, physical activity, diabetes mellitus, hypertension and general health status

*Q* quintile, *HR* hazard ratio, *CI* confidence interval

Participants in different retinal age gap quintiles had different survival distributions of incident stroke based on log-rank test (*P* < 0.001). After adjusting for confounding factors, participants whose retinal age gaps were in the fifth quintile had significantly higher risks of stroke compared to those whose retinal age gaps were in the first quintile (HR = 2.37, 95% CI: 1.37–4.10, *P* = 0.002). However, participants with retinal age gaps in the second, third and fourth quintiles had comparable risks of stroke compared to those with retinal age gaps in the first quintile. (HR = 1.16, 95% CI: 0.80–1.69, *P* = 0.433; HR = 1.10, 95% CI: 0.72–1.70, *P* = 0.660; HR = 1.29, 95% CI: 0.78–2.1, *P* = 0.323, respectively). A trend in the prediction accuracy of stroke across different quintiles of retinal age gaps (HR _trending_ = 1.17, 95% CI: 1.03–1.33, *P* = 0.016) was noted.

### Sensitivity analysis

Results comparable to those of the main analysis were noted when the age-squared term was included in the final model (Additional file 1: Table S2). We also found that retinal age acceleration residual was significantly associated with incident stroke (Additional file 1: Table S3).

### Predictive value of retinal age in stroke

**Fig. 1:**
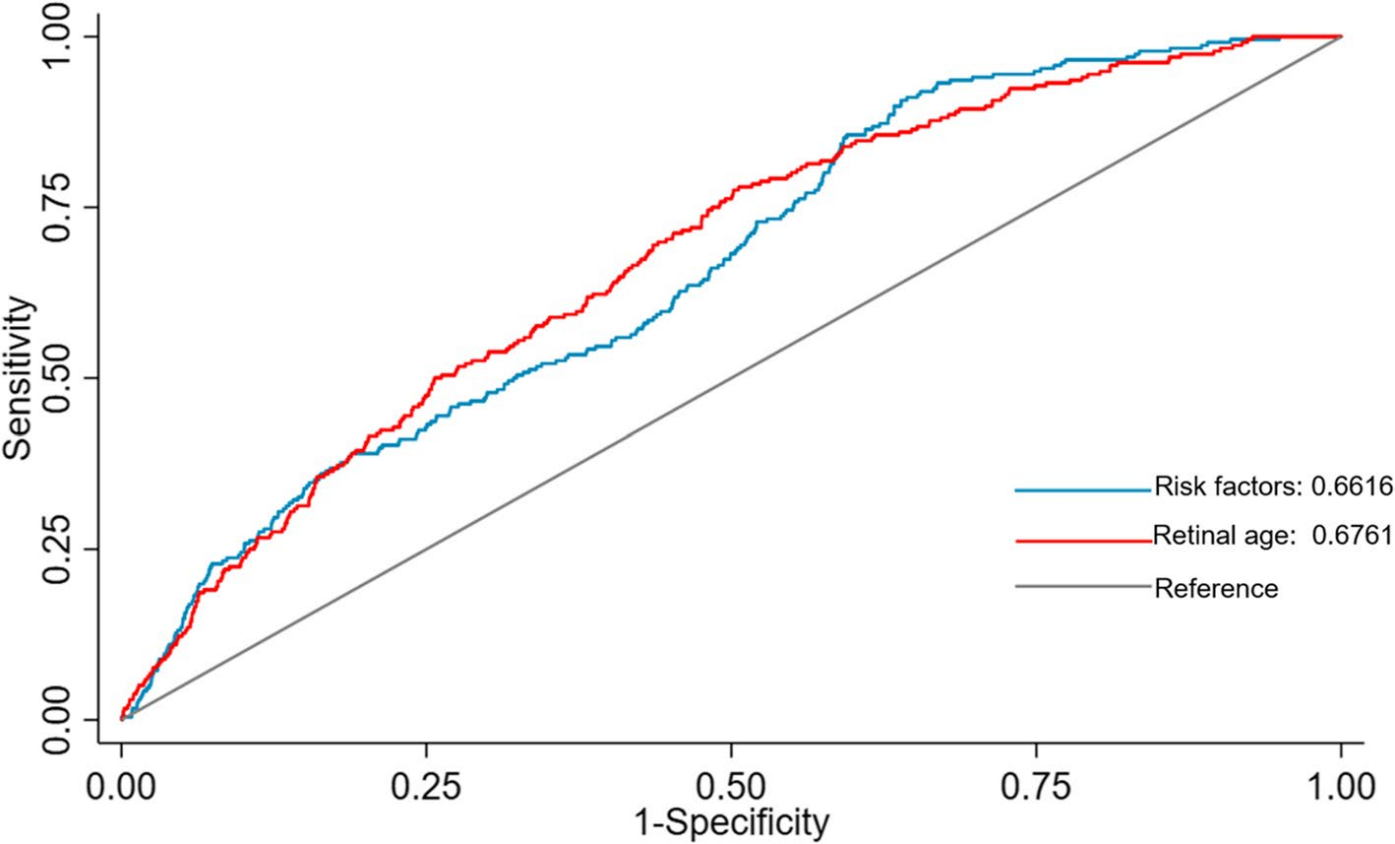
The predictive value of retinal age-based model and risk factor-based model in 10-year stroke risk. The AUC of retinal age-based model was 0.676 (95% CI: 0.644–0.708), which is slightly higher than the risk factor-based model (0.662, 95% CI: 0.630–0.693), but no significant difference was found (*p*=0.511)

The predictive value of a well-established risk factor-based model (including age, gender, smoking status, history of diabetes, systolic blood pressure, and total cholesterol to HDL-cholesterol ratio) and retinal age-based model in the prediction of 10-year stroke risk was shown in Fig. 1. The AUCs of the retinal age-based model was slightly higher than that of risk factor-based model (0.676, 95% CI: 0.644–0.708; 0.662, 95% CI: 0.630–0.693), while the difference did not reach significance (*p*=0.511).

## Discussion

In this large prospective cohort study, we found that retinal age gap was associated with an increased risk of incident stroke independent of classic stroke risk factors. Each 1-year increase in retinal age gap contributed to a 4% increase in the risk of stroke. Compared to participants with retinal age gaps in the lowest quintile, those with retinal age gaps in the highest quintile had a 2.37-fold increase in the risk of incident stroke. Further, retinal age demonstrated a predictive value compared to well-established risk factor-based model, indicating that accelerated biological ageing manifested as increased retinal age gaps could be a predictor for the development of stroke.

Our study linked the retinal age gap, as a holistic measure of age-related neuronal and vascular changes, with the risk of stroke and demonstrated the predictive value of retinal age in future risk of stroke. This is the first study to prove the concept of the association between the retinal age gap and risk of stroke, the observed association between ageing features or pathological changes observed in the retina and the risk of stroke is supported by previous studies [19, 28–30]. It was found in cross-sectional studies that patients with stroke have decreased central retinal artery equivalent (CRAE) and arteriolar-to-venule ratio (AVR) and are more likely to have focal arterial narrowing, arteriovenous nicking (AV nicking) and retinopathies [31–34]. As well, in cohort studies, multiple retinal vessel measurements such as tortuosity and fractal dimension, hypertensive or diabetic retinopathies, and retinal vessel occlusion were found to be significantly associated with future stroke risk [17–21].

In addition, our findings that the predictive value of retina age alone is comparable to the well-established clinical risk factor-based model suggested the retinal age can provide clues informative of end-organ damage in the eye. There are previous studies investigating the predictive values of retinal measurements or features which are supportive to our findings. Mitchell et al. found retinal microvascular signs (e.g. retinopathy, AV nicking and focal arteriolar narrowing) could predict incident stroke events independent of other risk factors of stroke [29]. Cheung et al. found that the accuracy of stroke prediction was improved by incorporating retinal microvascular signs, including retinopathy and widening of venous calibre, into the traditional risk factors [35]. Compared to previous studies which relied on grading or extraction of specific retinal features or pathologies, or definitions of retinal diseases, our concept of retinal age provides a different angle to examine the association between the retina and stroke. The present DL model could learn the appropriate predictive features based on a large sample of fundus images automatically and integrate all of the features from one image to generate an instant estimate of the biological age to predict stroke, which holds the advantage of less bias, the ability to capture the implicit retinal features and comprehensively describe the ageing characteristics that can be reflected through the retina.

Our findings also add new evidence to the limited body of knowledge about biological age and stroke. Brain age, a measurement of biological age, was reported to have an association with stroke [14, 36]. Biological age, estimated by DNA methylation, has also been associated with the risk of stroke and prognosis [12, 15, 37, 38]. The brain age was less accurate compared to the retinal age, when comparing the prediction accuracies in biological age [10, 26]. Notably, most studies about the brain age and stroke were cross-sectional studies, thus the accuracy of the brain age in predicting stroke was not clear. Although DNA methylation has been reported to independently predict the risk of stroke [38, 39], retinal age has the advantage of being non-invasive and cost-effective.

Several mechanisms may explain our findings. Firstly, the retina could act as a “window” to the body. The retinal and cerebral circulation undergo similar changes in morphology, blood flow and metabolic demand during the ageing process [22]. This might be explained by the common mechanisms underlying vascular ageing, such as endothelial dysfunction, oxidative stress and inflammation [40, 41]. Secondly, well-established risk factors of stroke could manifest themselves as retinal features on fundus images. For example, hypertension, as a risk factor of stroke, could cause retinal arteriolar changes in an early stage, including arteriolar narrowing and AV nicking, which were reported to be associated with stroke events [29]. Thirdly, growing evidence has showed that retinal diseases have the potential to predict stroke such as diabetic retinopathy and retinal artery and venous occlusion [20, 42, 43]. This is consistent with our findings that the attention maps of the DL models focused on the areas surrounding the retinal vessels, highlighting the important role of pathophysiological changes in retinal vasculature in predicting incident stroke [26].

### Future directions and outlook

Future studies are still needed to improve the DL algorithm and investigate into its real-world application and extrapolate its clinical value. First of all, external validations using other datasets comprising populations of different demographic features, such as older age and different ethnicities, and even using different retinal imaging modalities as input, such as optic coherence tomography data, are needed to improve the generalizability of the model and refine the algorithm for more large-scale application. In addition, given the heterogeneity of stroke types and pathogenesis such as ischaemic stroke and hemorrhagic stroke, subgroup analyses for stroke subtypes are needed to further streamline the use of retinal age gap in the prediction of stroke and provide clues to disentangle different mechanisms of stroke prediction using retinal images by the DL algorithm.

Our findings have the potential to provide several important public health and clinical implications after future refinement and clinical proof-of-concept studies. Retinal age gap has great potential to be used as a novel screening tool for individuals at high risks of stroke. Compared with those well-established prediction tools based on classic risk factors, such as the Framingham Stroke Risk Score [44], the retinal age gap assessment is characterized by convenient, non-invasive and cost-effective features, showing an enormous potential for further applications. For example, this DL algorithm may be incorporated into mobile devices, making the assessment of the retinal age gap and risk of stroke more easily accessible. This could facilitate the early referral of patients at high risks of stroke for preventative and therapeutic interventions to reduce the burden of stroke for individuals and society as a whole.

Despite the strengths of the present study including the large sample size, the long follow-up duration, comprehensive adjustments for confounding factors and standard acquisition of fundus images, several limitations should be considered. First, no external validation was performed for the retinal age prediction model, given that we have currently no access to external datasets with long-term follow-up and enough stroke events. Secondly, the UK Biobank cohort is comprised of relatively young participants (within the age range of 40–69 years). Moreover, the quality check tends to exclude the images of participants with older age. Considering the high risk of stroke in the more elderly population, our findings may be subject to generalizability. Further studies are needed to investigate the association between the retinal age gap and incident stroke in the elderly population. Nevertheless, the limited generalizability would not affect the association between the retinal age gap and stroke [45]. Thirdly, due to the low incidence rate of stroke and the lack of data on the causes of stroke, further subgroup analyses could not be conducted. Fourthly, due to the observational design of the study, we could not infer the causal effect of the ageing features of the retina on incident stroke. Lastly, we could not completely exclude the possibility of residual confounding.

## Conclusions

We found that retinal age gap, estimated based on retinal images, was associated with incident stroke. As a novel biomarker for stroke risk, the retinal age gap has great potential in enabling more accurate, accessible, efficient, cost-effective and non-invasive stroke screening. Further studies are warranted to confirm our findings in different populations and to explore the effect of the dynamic changes of retinal age gap in predicting risks of stroke.

## Supplementary Information

**Additional file 1: Table S1.** Baseline Characteristics Stratified by Participants with no/one/two good images that passed quality check. **Table S2.** Association Between Retinal Age Gap with Incident of Stroke additionally adjusted for age squared. **Table S3.** Association Between Retinal Age Acceleration Residual with Incident of Stroke.

## Data Availability

The dataset analysed during the present study is available in the UK Biobank (https://www.ukbiobank.ac.uk/). These data are available from the corresponding author on reasonable request and with permission of UK Biobank.

## Abbreviations

HR: Hazard ratio
CI: Confidence interval
AUC: Receiver-operating-characteristic curve
CNS: Central nervous system
DL: Deep learning
NHS: National Health Service
OCT: Optical coherence tomography
MAE: Mean absolute error
ICD: International Classification of Diseases
HDL: High-density lipoproteins
TDI: Townsend deprivation indices
BMI: Body mass index
SD: Standard deviation
IQR: Interquartile range
CRAE: Central retinal artery equivalent
AVR: Arteriolar-to-venule ratio
AV nicking: Arteriovenous nicking
