# Supplementary material for: Retinal age gap as a predictive biomarker of stroke risk
Source: BMC Med. 2022 Nov 30;20:466. doi: 10.1186/s12916-022-02620-w (PMC9710167; doi:10.1186/s12916-022-02620-w)
Supplement: Supplementary file 1 — Additional file 1: Table S1. Baseline Characteristics Stratified by Participants with no/one/two good images that passed quality check. Table S2. Association Between Retinal Age Gap with Incident of Stroke additionally adjusted for age squared. Table S3. Association Between Retinal Age Acceleration Residual with Incident of Stroke. [file 12916_2022_2620_MOESM1_ESM.docx]

Supplement table 1. Baseline Characteristics Stratified by Participants with no/one/two good images that passed quality check.

| **Baseline Characteristics** | **Overall** | **No-Good Image Group** | **One-Good Image Group** | **Two-Good Image Group** | ***P* value** |
| --- | --- | --- | --- | --- | --- |
| *N* | 67,351 | 20,382 | 13,769 | 33,200 | - |
| Age, mean (SD), yrs | 56.7 (8.15) | 58.8 (7.57) | 57.3 (7.97) | 55.1 (8.23) | **<0.001** |
| Gender, No. (%) | | | | | |
| Female | 36,747 (54.6) | 10,814 (53.1) | 7,325 (53.2) | 18,608 (56.1) | **<0.001** |
| Male | 30,604 (45.4) | 9,568 (46.9) | 6,444 (46.8) | 14,592 (43.9) |  |
| Ethnicity, No. (%) | | | | | |
| White | 60,568 (89.9) | 17,081 (83.8) | 12,457(90.5) | 31,030 (93.5) | **<0.001** |
| Others | 6,784 (10.1) | 3,301 (16.2) | 1,313 (9.54) | 2,170 (6.54) |  |
| Deprivation index, mean (SD) | -1.02 (3.00) | -0.87 (3.09) | -1.02(3.01) | -1.10(2.93) | **<0.001** |
| Education level, No. (%) | | | | | |
| College/university | 23,752 (35.3) | 6,715 (33.0) | 4787 (34.8) | 12,250 (36.9) | **<0.001** |
| Others | 43,600 (64.7) | 13,667 (67.0) | 8983 (65.2) | 20,950 (63.1) |  |
| Smoking status, No. (%) | | | | | |
| Never | 37,345 (55.8) | 10,879 (53.9) | 7,541 (55.1) | 18,925 (57.3) | **<0.001** |
| Former/current | 29,567 (44.2) | 9,312 (46.1) | 6139 (44.9) | 14,116 (42.7) |  |
| Drinking status, No. (%) | | | | | |
| Never | 3,300 (4.92) | 1,236 (6.10) | 691 (5.04) | 1,373 (4.15) | **<0.001** |
| Former/current | 63,777 (95.1) | 19,025 (93.9) | 13,030 (95.0) | 31,722 (95.8) |  |
| Obesity, No. (%) | | | | | |
| No | 50,884 (75.9) | 14,920 (73.6) | 10278 (75.1) | 25,686 (77.7) | **<0.001** |
| Yes | 16,126 (24.1) | 5,351 (26.4) | 3,416 (24.9) | 7,359 (22.3) |  |
| Meeting PA recommendation, No. (%) | | | | | |
| No | 9,681 (17.6) | 2,926 (17.9) | 2,051 (18.2) | 4,704 (17.1) | **0.016** |
| Yes | 45,468 (82.4) | 13,437 (82.1) | 9,225 (81.8) | 22,806 (82.9) |  |
| History of diabetes, No. (%) | | | | | |
| No | 63,220 (93.9) | 18,665 (91.6) | 12,901 (93.7) | 31,654 (95.3) | **<0.001** |
| Yes | 4,132 (6.13) | 1,717 (8.42) | 869 (6.31) | 1,546 (4.66) |  |
| History of hypertension, No. (%) | | | | | |
| No | 17,679 (26.3) | 4,678 (22.9) | 3,444 (25.0) | 9,557 (28.8) | **<0.001** |
| Yes | 49,673 (73.7) | 15,704 (77.1) | 10,326 (75.0) | 23,643 (71.2) |  |
| General health status, No. (%) | | | | | |
| Excellent/good | 48,895 (73.1) | 14,333 (70.9) | 9,898 (72.4) | 24,664 (74.7) | **<0.001** |
| Fair/poor | 18,012 (26.9) | 5,870 (29.1) | 3,780 (27.6) | 8,362 (25.3) |  |

SD = standard deviation; PA = physical activity;

| Supplement table 2. Association Between Retinal Age Gap with Incident of Stroke additionally adjusted for age squared. | | | | |
| --- | --- | --- | --- | --- |
|  | **Model III** | | **Model IV** | |
| **Retinal age gap** | **HR (95% CI)** | **P value** | **HR (95% CI)** | **P value** |
| Retinal age gap, per one age (yrs) | 1.05 (1.02-1.09) | **0.004** | 1.05 (1.01-1.09) | **0.016** |
| Retinal age gap | | | | |
| Q1 | 1 [Reference] | - | 1 [Reference] | - |
| Q2 | 1.29 (0.93-1.77) | 0.124 | 1.23 (0.84-1.79) | 0.291 |
| Q3 | 1.15 (0.78-1.69) | 0.478 | 1.20 (0.77-1.87) | 0.419 |
| Q4 | 1.57 (1.01-2.43) | **0.045** | 1.43 (0.85-2.42) | 0.18 |
| Q5 | 2.13 (1.29-3.52) | **0.003** | 2.51 (1.43-4.41) | **0.001** |
| Q = quintile; HR = hazard ratio; CI = confidence interval. | | | | |
| Model III adjusted for age, age squared, gender, and ethnicity (Model I +age squared). | | | | |
| Model IV adjusted for covariates in Model III + deprivation, education level, smoking status, drinking status, obesity, physical activity, diabetes mellitus, hypertension and general health status (Model II + age squared). | | | | |

| Supplement table 3. Association Between Retinal Age Acceleration Residual with Incident of Stroke. | | | | |
| --- | --- | --- | --- | --- |
|  | **Model I** | | **Model II** | |
| **Retinal age acceleration residual** | **HR (95% CI)** | **P value** | **HR (95% CI)** | **P value** |
| Retinal age acceleration residual, per one age (yrs) | 1.05 (1.01-1.08) | **0.006** | 1.04 (1.00-1.08) | **0.028** |
| Retinal age acceleration residual | | | | |
| Q1 | 1 [Reference] | - | 1 [Reference] | - |
| Q2 | 1.23 (0.82-1.83) | 0.32 | 1.15 (0.72-1.83) | 0.563 |
| Q3 | 1.24 (0.83-1.86) | 0.297 | 0.90 (0.55-1.47) | 0.675 |
| Q4 | 1.27 (0.85-1.90) | 0.246 | 1.17 (0.74-1.86) | 0.493 |
| Q5 | 1.85 (1.26-2.71) | **0.002** | 1.69 (1.09-2.62) | **0.018** |
| Q = quintile; HR = hazard ratio; CI = confidence interval. | | | | |
| Model I adjusted for age, gender, and ethnicity. | | | |  |
| Model II adjusted for covariates in Model I＋deprivation, education level, smoking status, drinking status, obesity, physical activity, diabetes mellitus, hypertension and general health status. | | | | |
